# Supplementary material for: Wildfire legacies on pyrogenic carbon stocks in Amazonian peatlands
Source: Commun Earth Environ. 2025 Aug 19;6(1):678. doi: 10.1038/s43247-025-02674-7 (PMC12360956; doi:10.1038/s43247-025-02674-7)
Supplement: Supplementary file 2 — Supplementary Information [file 43247_2025_2674_MOESM2_ESM.pdf]

Supplementary Information for

Wildfire legacies on pyrogenic carbon stocks in Amazonian peatlands

Authors: Yuwan Wang[1,2*], Angela Gallego-Sala[1], Michael I. Bird[3], Patrick Moss[2,4], Hamish A. McGowan[2], Juan C. Benavides[5], Euridice N. Honorio Coronado[6], Ted R. Feldpausch[1*]

1 Department of Geography, University of Exeter, Exeter, UK
2 School of the Environment, University of Queensland, Brisbane, Australia
3 College of Science and Engineering and ARC Centre of Excellence for Indigenous and Environmental Histories and Futures, James Cook University, Cairns, Australia
4 School of Earth & Atmospheric Sciences, Queensland University of Technology, Brisbane, Australia
5 Department of Ecology and Territory, Pontificia Universidad Javeriana, Bogotá, Colombia
6 Royal Botanic Gardens, Kew, Richmond, London, UK

This file contains:

Supplementary Discussion

Supplementary Figures 1-7

Supplementary Tables 1-5

Supplementary Discussion:

Detailed descriptions of the uncertainties and limitations in PyC stock calculations

Considering the small number of PyC observations from peatlands located mainly in the northwestern Amazon Basin in this study, it is important to note that our estimate of PyC stock for the whole basin relies on two main assumptions. First, we assumed TOC is equally distributed across the age groups; this assumption is based on the nature of peat formation, where, unless exogenous mineral materials are entering the system, TOC tends to present a relatively homogeneous profile with depth. With a decreasing but insignificant trend in TOC with age in our study sites (slope: -0.0013%C ka^{-1}), we believe this assumption is acceptable. This assumption is further supported by a previous study that compiled a large number of high-latitude northern peatland observations (N = 3,741), which showed that carbon content is uniformly distributed over depth in peatlands during the Holocene period [1].

Secondly, we determined the general age of peat inception in the basin by applying a median peat basal age of 4,450 cal yr BP, which is lower than either the weighted average age of 6,202 cal yr BP or average age of 5,907 cal yr BP from our compilation in Supplementary Table 2. The values are 7,530, 7,489 and 7,732 cal yr BP for median, average and weighted average ages for 1,097 peatlands of global compilation [2] and these three values in this global compilation only show a small deviation of ~250 years. It is unclear whether Amazonian peatland values will follow a similar pattern to the global peat initiation ages as the three values (median, average and weighted average ages) in our current compilation are not close to each other. Given that very old dates in our compilation contributed substantially to the age distribution, we selected the median value in the calculations to provide a conservative estimate of PyC stock.

An additional concern in this study is that the estimation of PyC stock is based on its relationship with age instead of depth. This approach aligns with a previous PyC stock estimation in northern peatlands, which also used age as the predictor [3]. Using age also facilitates the direct application of equations and allows linking to climatic settings that could affect PyC formation, over similar timescales. Additionally, PyC estimation based on the PyC-depth relationship yields 0.32±0.37 Pg of PyC for basin-wide peatlands (Supplementary Figure 6),

representing only 0.7% of TOC stock in Amazonian peatlands. This is much lower than the 1.2% average in our observations. Therefore, the age-based estimation is preferred in this study.

Several uncertainties and limitations beyond the scope of this study were not fully addressed. Caution needs to be exercised when comparing results across different PyC quantification methods because there exist non-systematic offsets between current methods [4, 5]. For example, the HyPy method used in this study has been suggested to report higher values in environmental matrices [6], such as soils, compared to the chemo-thermal oxidation method CTO-375, which is a widely used method to quantify PyC after removing inorganic carbonates by acidification and labile organic matter via combustion [7]. Although considering this methodological difference, our HyPy-derived PyC/TOC still indicates lower values in peatlands compared to reported values in Amazonian forest soils.

The peat map of the Amazon Basin is a large source of uncertainty, which is not considered in this study, and an accurate map of the whole region does not exist. Peat maps are better developed for certain regions where field campaigns have been more extensive [8, 9]. For other areas, the accuracy of the maps may be lower. For example, Melton, Chan [10] questioned the possibility of extensive peat formation in seasonally flooded savannas and white sand ecosystems in the basin, and reduced previous estimates of area by about half [11]. The first field data-driven peatland map for the basin has recently been published [12] and would provide a lower estimate of 0.40 Pg of PyC (based on a different boundary of the Amazon Basin from our study). There is still a need for more ground-truthing data to verify the accuracy of these different peat maps, especially for remote areas where accessibility is restricted. Additionally, the coupling between PyC/TOC and peat inception ages remains uncertain; further efforts to fill the above data gaps in Amazonian peatlands are needed to accurately assess our estimated PyC stock.

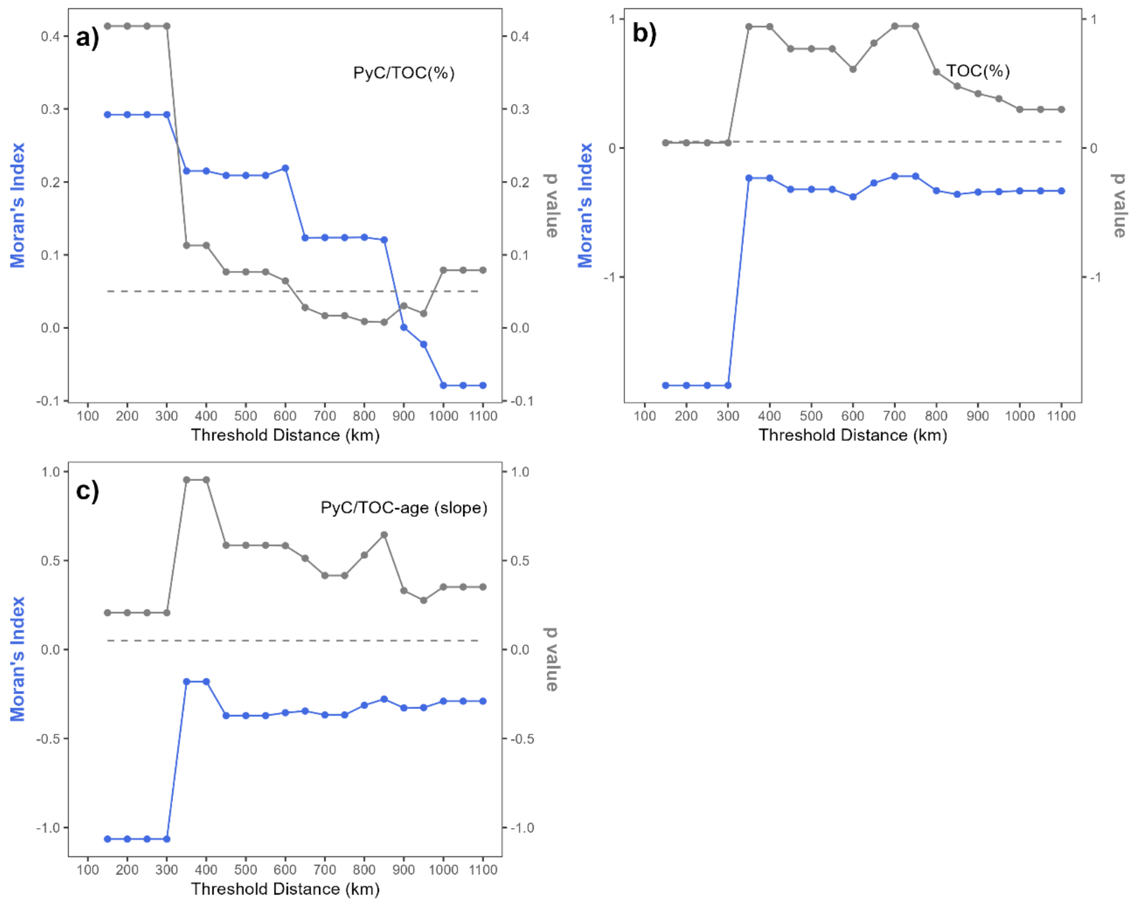

Supplementary Figure 1 Spatial autocorrelation analysis of PyC/TOC, TOC, and PyC/TOC-age in study sites. Global Moran's index and related p-values for spatial autocorrelation in (a) PyC/TOC, (b) TOC and (c) the relationship between PyC/TOC and age (slope from simple linear regression model) based on different thresholds of Euclidean distance in Global Moran's I test. Dashed line marks the statistical significance threshold ($p = 0.05$)

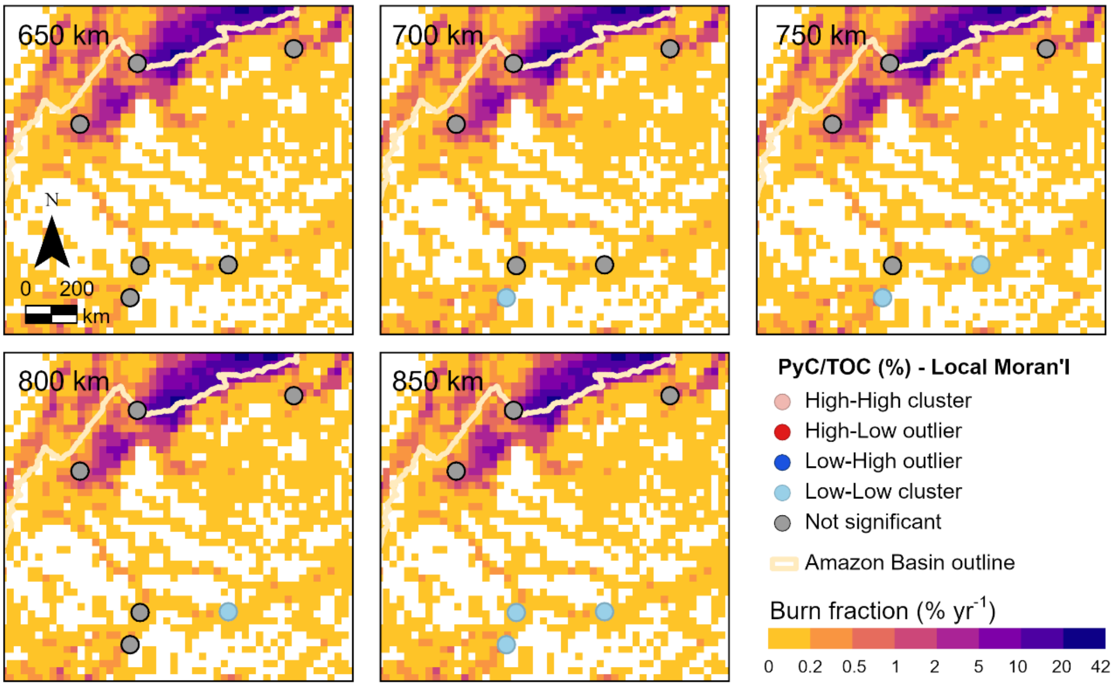

Supplementary Figure 2 Local Moran's I maps of PyC/TOC at Euclidean distance thresholds where Global Moran's I was significantly positive. This map is based on the Global Fire Emissions Database with small fires (GFEDs) burned fraction over the period of 1997-2016 [13].

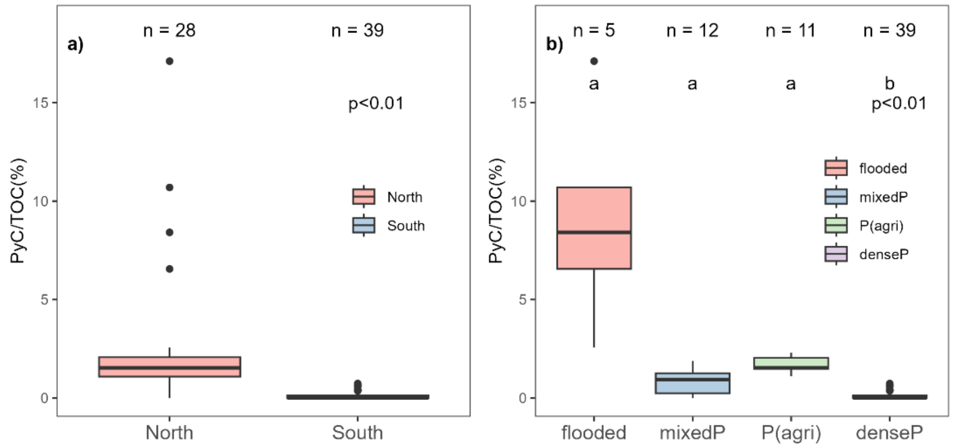

Supplementary Figure 3 Boxplot of percentage of pyrogenic carbon in total organic carbon across site locations and types. (a) site location (North: INI, PLL and CAQ; South: QUI, JEN and CAN); (b) site type (flooded-flooded forest: INI; mixed P-mixed palm swamp: CAQ; P(agri): palm swamp surrounded by agricultural land: PLL and denseP-*Mauritia*-dominated palm swamp: QUI, JEN and CAN). $p<0.01$ from Kruskal-Wallis test for site locations and types. The different letters on the top in (b) indicate significant differences (Dunn's *post hoc* test).

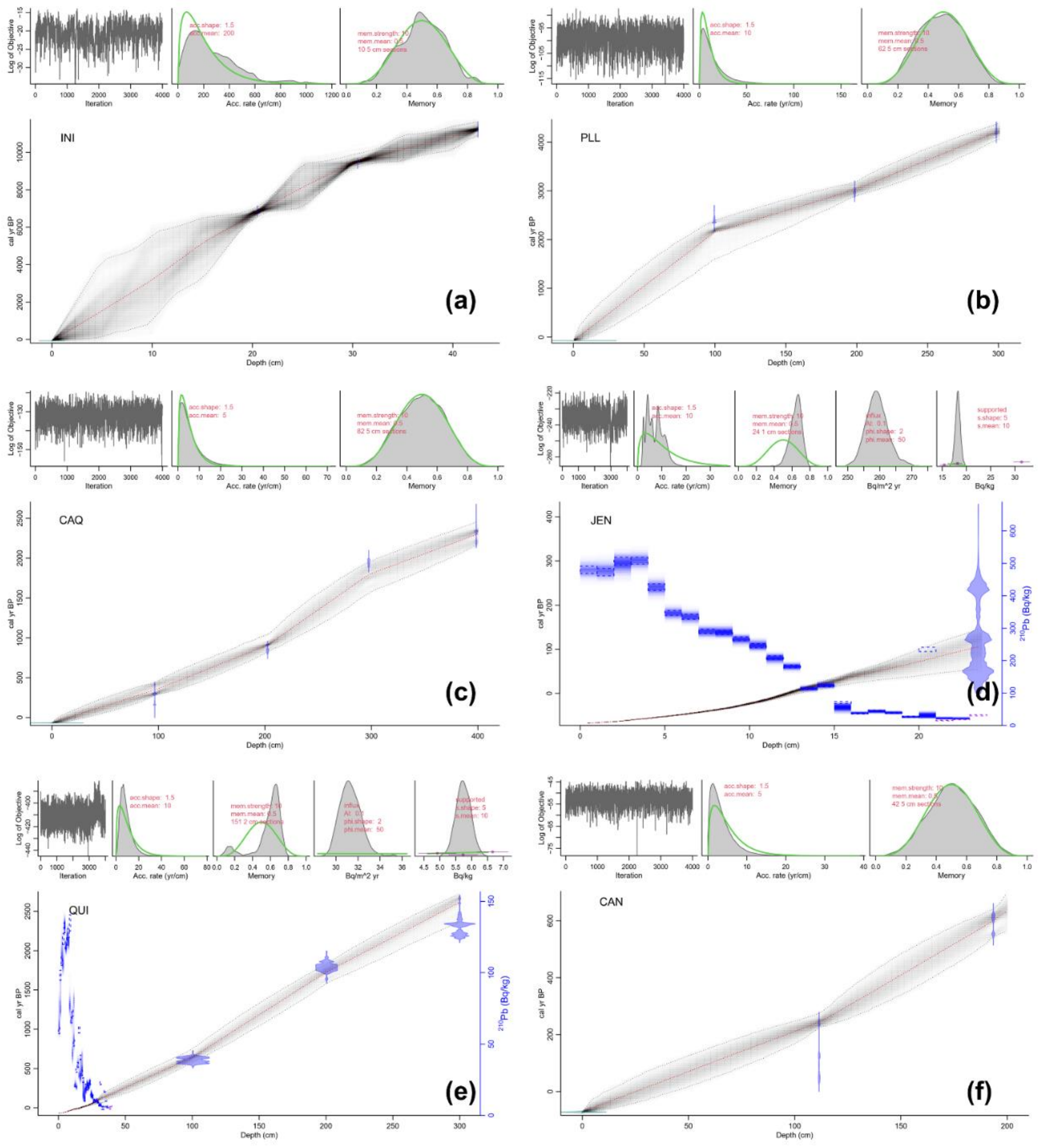

Supplementary Figure 4 Age-depth models of study sites using the *rbacon* [14] and *rplum* [15]. (a) Inírida (INI), (b) Puerto Lleras (PLL), (c) Caquetá (CAQ), (d) Jenaro Herrera (JEN), (e) Quistococha (QUI) and (f) Cananguchal (CAN).

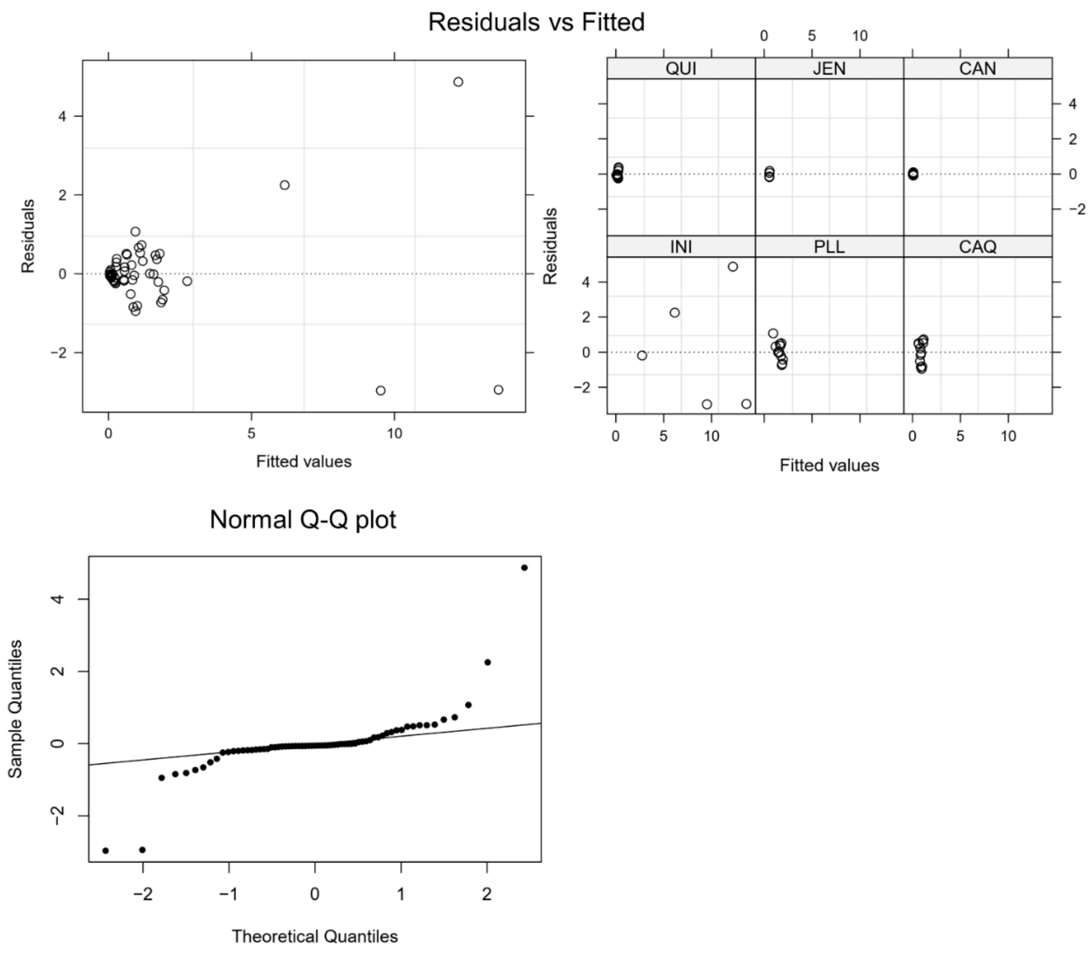

Supplementary Figure 5 The residual vs fitted plot (all data and grouped by different sites) and Normal Q-Q plot for data shown in Figure 2.

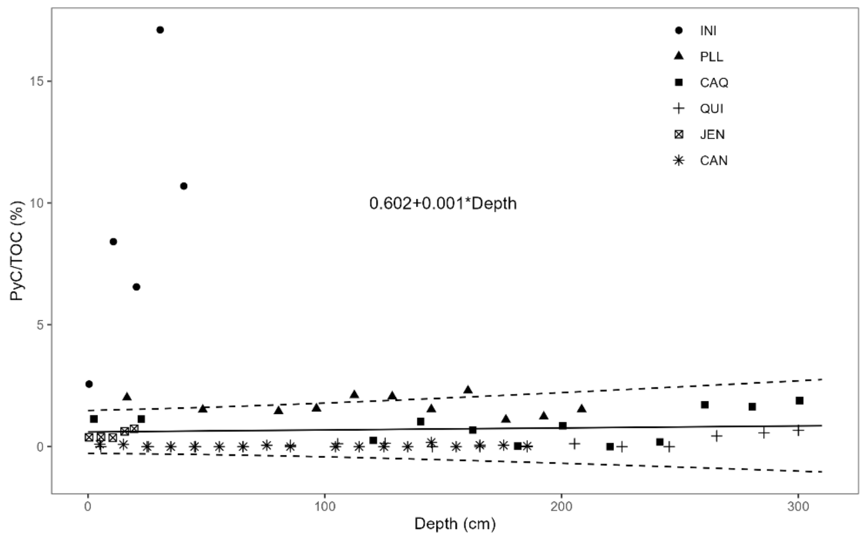

Supplementary Figure 6 The relationship between the percentage of pyrogenic carbon in total organic carbon and peat depth in Amazonian peatlands (N=67). The shape of data points indicates different peat cores. The solid line is the best model derived from a linear mixed-effects model with a random slope (equation insert). Dashed lines indicate a 95% confidence interval. This gives the estimation of 0.32±0.37 Pg PyC for basin-wide peatland.

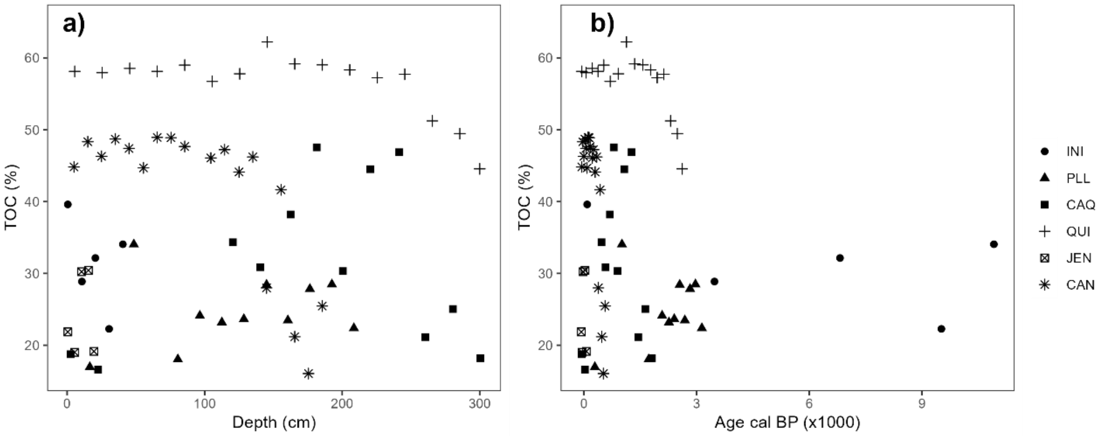

Supplementary Figure 7 The relationship between TOC and (a) depth and (b) age in study sites. The shape of data points indicates different peat cores.

Supplementary Table 1 Dataset of peat depth, dry bulk density and carbon content in Amazonian peatlands available in the literature and from this study. Elevation is obtained from SRTM Digital Elevation Data Version 4 [16].

ID	Site	Lat (°)	Lon (°)	Elevation (m)	Country	No.	Peat depth (cm)	No.	BD (g cm^{-3})	C (%)	Ref.
1	Buena Vista	-4.25	-73.20	97	Peru	7	212				[8, 17]
2	Charo	-4.27	-73.26	104	Peru	6	126				[8, 17]
3	Ex Petroleros	-4.08	-73.46	109	Peru	4	110				[8, 17]
4	Fundo Junior	-4.10	-73.32	116	Peru	13	268				[8, 17]
5	Las Brisas	-3.80	-73.30	92	Peru	1	190	1	0.12	49.40	[8, 18]
6	Pebas	-3.38	-71.84	81	Peru	2	55				[8, 17]
7	Primavera	-3.37	-71.81	101	Peru	10	62				[8, 17]
8	Quistococha	-3.84	-73.32	103	Peru	12	250	22	0.09	47.37	[8, 17, 18]
9	Rio Napo 1a	-3.20	-73.20	109	Peru	1	320				[8]
10	Rio Napo 1b	-3.20	-73.30	121	Peru	1	392				[8]
11	San Jorge	-4.06	-73.19	107	Peru	16	291	22	0.11	44.29	[8, 17]
12	San Nicolas	-3.65	-71.70	96	Peru	10	128				[8, 17]
13	Santa Rosa	-3.73	-73.16	103	Peru	6	102				[8, 17]
14	Tarapoto	-3.79	-73.41	97	Peru	4	143				[8, 17]
15	Cuninico	-4.80	-75.20	128	Peru	8	288				[8, 19]
16	Lagunas	-5.20	-75.60	128	Peru	5	77	4	0.12	44.51	[8, 19]
17	Nueva Alianza	-4.70	-75.40	119	Peru	5	260	8	0.05	45.60	[8, 19]
18	Ollanta	-4.50	-74.90	104	Peru	41	227				[8, 20]
19	Parinari	-4.60	-74.50	118	Peru	1	111				[8, 20]
20	Pobre Cocha	-4.80	-74.30	114	Peru	1	55	1	0.22	33.50	[8, 18]
21	Roca Fuerte	-4.40	-74.80	108	Peru	8	429	14	0.07	51.60	[8, 19]
22	San Martin	-4.80	-74.40	123	Peru	1	39	1	0.27	22.50	[8, 18]
23	San Miguel	-4.70	-74.20	120	Peru	1	35	1	0.23	19.50	[8, 18]
24	San Roque	-4.50	-74.60	119	Peru	6	316	15	0.11	41.64	[8, 19]
25	San Roque 4	-4.50	-74.60	119	Peru	8	217				[8]
26	Santa Rita	-4.60	-74.40	98	Peru	1	90				[8, 20]
27	Santa Rosa de Lagarto 1	-4.40	-74.70	117	Peru	6	156				[8]

28	Santa Rosa de Lagarto 2	-4.40	-74.70	117	Peru	8	258				[8]
29	Santa Rosa de Lagarto 3	-4.40	-74.60	115	Peru	10	349				[8]
30	Saramuro	-4.70	-74.90	121	Peru	1	65				[8, 20]
31	Shiringal	-4.80	-74.30	114	Peru	1	34	1	0.31	27.50	[8, 18]
32	Tacshacocha	-4.90	-74.30	119	Peru	6	67	2	0.07	33.20	[8, 19]
33	Veinte de Enero	-4.70	-73.80	105	Peru	41	194				[8, 20]
34	W Maranon 3	-4.90	-75.50	127	Peru	5	109				[8]
35	Aucayacu	-3.90	-74.40	121	Peru	9	500	23	0.11	48.93	[8, 19]
36	Avispa Cocha	-4.20	-74.40	115	Peru	1	225	1	0.13	39.60	[8, 18]
37	Chanchari	-4.00	-74.40	120	Peru	1	280	1	0.11	52.70	[8, 18]
38	Cristo Rey/Bellavista	-4.00	-74.30	119	Peru	11	203	2	0.09	44.66	[8]
39	Cuchara 1	-3.80	-74.80	142	Peru	15	263	6	0.08	53.63	[8]
40	Cuchara 2	-3.80	-74.80	142	Peru	9	255	9	0.07	49.72	[8]
41	Florida	-3.80	-74.50	128	Peru	13	153	11	0.09	43.05	[8]
42	Llanque	-4.20	-74.40	115	Peru	1	118	1	0.13	40.00	[8, 18]
43	Malvinas	-4.00	-74.40	120	Peru	12	313	9	0.10	46.46	[8]
44	Miraflores	-4.40	-74.10	118	Peru	9	249	10	0.08	49.68	[8, 19]
45	Monteverde 1	-4.10	-74.40	116	Peru	9	117	3	0.11	46.15	[8]
46	Monteverde 2	-4.20	-74.40	115	Peru	11	145	1	0.15	28.65	[8]
47	Monteverde 3	-4.20	-74.40	115	Peru	1	188	1	0.16	26.60	[8, 18]
48	Nueva York	-4.40	-74.30	116	Peru	10	413	15	0.06	52.19	[8, 19]
49	Nueva York 2	-4.40	-74.30	116	Peru	1	240	1	0.11	44.40	[8, 18]
50	Nueva York 3	-4.30	-74.40	108	Peru	1	100				[8]
51	Puerto Oriente	-3.70	-74.70	134	Peru	13	273	5	0.09	46.83	[8]
52	San Carlos	-3.80	-74.60	124	Peru	13	208	7	0.08	51.41	[8]
53	Sanango	-3.60	-74.60	124	Peru	12	41	3	0.05	40.12	[8]
54	BV de Maquia	-6.20	-74.70	128	Peru	7	118	5	0.09	38.49	[8, 19]
55	Maquıía	-6.30	-74.80	122	Peru	8	388	17	0.07	43.54	[8, 19]
56	Nuevo Encanto	-5.50	-74.50	115	Peru	4	126				[8, 19]
57	Riñón	-4.90	-74.00	100	Peru	9	356	8	0.06	49.13	[8, 17]

58	Tapiche Blanco 1	-5.70	-73.90	117	Peru	15	75				[8]
59	Tapiche Blanco 3	-5.80	-73.90	106	Peru	11	63				[8]
60	Tapiche Blanco 4	-5.70	-73.90	117	Peru	11	60				[8]
61	Tapiche Blanco 5	-5.70	-73.90	117	Peru	10	81				[8]
62	Victoria	-5.70	-74.60	112	Peru	7	30				[8, 19]
63	Los Amigos Aguajal	-12.56	-70.12	240	Peru	1	573	1	0.20	28.50	[21]
64	Cuatro Vientos	-14.52	-61.12	169	Bolivia	1	230				[22]
65	Marahuaka I.2	3.67	-65.43	2684	Venezuela	1	70				[23]
66	Huachama I.6	3.85	-65.73	1195	Venezuela	1	60				[23]
67	Cuao 2	5.05	-67.37	1060	Venezuela	1	87				[23]
68	Cuao 7	4.92	-67.18	936	Venezuela	1	60	1	0.16		[23]
69	Cuao 11	4.98	-67.30	798	Venezuela	1	43				[23]
70	Cuao 12	4.90	-67.35	1264	Venezuela	1	150				[23]
71	Cuao 13	5.00	-67.43	632	Venezuela	1	45				[23]
72	Maigualida 1	5.57	-65.22	2119	Venezuela	1	130				[23]
73	Maigualida 2	5.53	-65.15	1607	Venezuela	1	35				[23]
74	Acopán-tepui	5.18	-62.08	2152	Venezuela	1	140				[24, 25]
75	Acopán-tepui	5.18	-62.08	2152	Venezuela	1	190				[24, 25]
76	Auyán-tepui	5.92	-62.63	1353	Venezuela	1	168				[24, 25]
77	Churí-tepui	5.25	-62.02	2381	Venezuela	1	83				[24, 25]
78	Divina Pastora pond	4.70	-61.07	852	Venezuela	1	200				[24, 25]
79	Guaiquinima	5.90	-63.70	1465	Venezuela	1	150				[24, 25]
80	Guaiquinima	5.90	-63.70	1465	Venezuela	1	160				[24, 25]
81	Quebrada Arapán	5.17	-61.10	1102	Venezuela	1	120				[24, 25]
82	Santa Cruz de Mapaurí	4.93	-61.10	866	Venezuela	1	240				[24, 25]
83	Santa Teresa pond	4.72	-61.08	856	Venezuela	1	160				[24, 25]
84	Toronó-tepui	5.27	-62.15	2159	Venezuela	1	200				[24, 25]
85	Toronó-tepui	5.27	-62.15	2159	Venezuela	1	150				[24, 25]
86	Urué	5.03	-61.17	852	Venezuela	1	200				[24, 25]

87	El Paují	4.47	-61.58	872	Venezuela	1	219				[26]
88	Lake Encantada	4.70	-61.07	852	Venezuela	1	393				[27]
89	Yasuni	-0.45	-76.62	230	Ecuador	1	252				[28]
90	Yasuni	-0.45	-76.62	230	Ecuador	1	266				[28]
91	Chimantá massif	5.32	-62.23	2226	Venezuela	1	170				[29]
92	Demeni	-0.65	-62.82	39	Brazil	7	100	8	0.14	47.60	[30]
93	Zalalá	-0.81	-62.72	31	Brazil	5	120	8	0.13	38.60	[30]
94	Daracuá	-0.44	-63.22	32	Brazil	4	150	7	0.14	42.60	[30]
95	Calibuqui	-0.59	-62.83	31	Brazil	3	30	2	0.31	31.00	[30]
96	Ucayali_1	-7.94	-74.60	157	Peru	8	136	8	0.12		[31]
97	Ucayali_2	-7.98	-74.54	149	Peru	8	150	8	0.19		[31]
98	Ucayali_3	-8.03	-74.50	161	Peru	14	312	14	0.12		[31]
99	Ucayali_4	-8.06	-74.46	162	Peru	10	148	10	0.13		[31]
100	Ucayali_5	-8.14	-74.39	164	Peru	9	503	9	0.13		[31]
101	Ucayali_8	-8.49	-74.07	162	Peru	4	112	4	0.10		[31]
102	Ucayali_9	-8.54	-74.63	163	Peru	4	100	4	0.08		[31]
103	Ucayali_10	-8.79	-74.06	160	Peru	15	259	15	0.11		[31]
104	Ucayali_13	-9.18	-74.10	183	Peru	8	92	8	0.13		[31]
105	Arguire	-12.64	-69.37	209	Peru	11	304				[32]
106	Bello Horizonte	-12.48	-69.05	198	Peru	15	108				[32]
107	Boli	-12.44	-68.69	176	Peru	22	185				[32]
108	CICRAa	-12.56	-70.09	286	Peru	194	316				[32]
109	CM2	-12.47	-70.26	259	Peru	15	85.9				[32]
110	Colorado	-12.46	-70.51	260	Peru	38	165.9				[32]
111	Huacho	-12.65	-69.41	197	Peru	20	275.3				[32]
112	Lagarto	-12.65	-69.78	209	Peru	60	234.8				[32]
113	Mercedes	-12.69	-69.46	204	Peru	41	245.9				[32]
114	SouthC	-12.65	-70.12	239	Peru	13	117.4				[32]
115	Mera	-1.48	-77.10	373	Ecuador	1	130				[33]
116	Ciega	3.07	-70.33	145	Colombia	1	610				[34]
117	Lake Gentry	-12.33	-68.87	248	Peru	1	43				[35]

118	Lake Parker	-12.14	-69.02	246	Peru	1	50				[35]
119	CaRío	1.13	-75.43	212	Colombia	11	174				[36]
120	Aña	1.13	-75.43	212	Colombia	5	140				[36]
121	Belleza	0.86	-75.25	201	Colombia	10	154				[36]
122	Panelera	0.86	-75.26	215	Colombia	10	66				[36]
123	Cananguchal	-3.81	-70.23	92	Colombia	1	200	1	0.12	40.77	this study
124	Caquetá	1.13	-75.43	212	Colombia	1	300	1	0.25	21.69	this study
125	Inírida	3.77	-67.93	99	Colombia	1	100	1	0.32	27.53	this study
126	Puerto lleras	3.26	-73.42	241	Colombia	1	290	1	0.36	28.42	this study
127	Quistococha	-3.83	-73.32	101	Peru	3	300	2	0.10	49.53	this study

Supplementary Table 2 Dataset of peat basal ages for Amazonian peatlands available in the literature and from this study. Elevation is obtained from SRTM Digital Elevation Data Version 4 [16].

ID	Site	Lat (°)	Lon(°)	Elevation (m)	Country	Core ID	Lab ID	Depth (cm)	Thickness (cm)	14C age	Dating material	Median prob. Cal yr BP	Ref.
1	Buena Vista	-4.25	-73.20	97	Peru	BQ15		304	8	1260 ± 25	peat	1152	[17]
2	Charo	-4.27	-73.26	104	Peru	CR15		172	8	715 ± 25	peat	654	[17]
3	Quistococha	-3.84	-73.32	103	Peru							2157.75	combine
		-3.84	-73.32	103	Peru	QT3		395	10	2300 ± 25	peat	2229	[17]
		-3.84	-73.32	103	Peru	QT-2010-1	UBA-18442	400.5	1	2161 ± 33	leaves and seeds	2116	[37]
		-3.84	-73.32	105	Peru	QT-2011-2	SUERC-44989	196		2155 ± 35	< 180 µm peat	2108	[38]
		-3.84	-73.32	104	Peru	QT-2011-3	SUERC-44990	178		2234 ± 37	< 180 µm peat	2232	[38]
		-3.83	-73.32	103	Peru	QT-2011-4	SUERC-44991	352		2253 ± 35	< 180 µm peat	2230	[38]
		-3.84	-73.32	103	Peru	QT-2011-5	SUERC-44992	226		1988 ± 37	< 180 µm peat	1910	[38]
		-3.84	-73.32	104	Peru	QT-2011-6	SUERC-44995	320		2061 ± 37	< 180 µm peat	1993	[38]
		-3.83	-73.32	101	Peru	QT-2011-7	SUERC-44996	262		2228 ± 35	< 180 µm peat	2233	[38]
		-3.83	-73.31	98	Peru	QT-2012-9	SUERC-54428	256		2324 ± 41	< 180 µm peat	2301	[38]
		-3.83	-73.31	100	Peru	QT-2012-10	SUERC-54429	180		2290 ± 41	< 180 µm peat	2231	[38]
		-3.85	-73.32	100	Peru	QT-2012-18	SUERC-54432	144		2059 ± 41	< 180 µm peat	1990	[38]
		-3.83	-73.32	101	Peru	QUI	SUERC-103541	300	1	2332 ± 37	macrofossil	2320	this study

4	Riñón	-4.90	-74.00	100	Peru	PS24		375	10	1700 ± 25	peat	1571	[17]
5	San Jorge	-4.06	-73.19	107	Peru							2537	combine
		-4.06	-73.19	107	Peru	SV24		565	10	2850 ± 35	peat	2939	[17]
		-4.06	-73.20	106	Peru	SJO-2010-1	SUERC-54422	239	2	2173 ± 41	< 180 μm peat	2135	[39]
6	Aucayacu	-3.94	-74.38	127	Peru							8224	combine
		-3.94	-74.38	127	Peru	AU8		735	10	7963 ± 35	peat	8806	[19]
		-3.94	-74.39	127	Peru		SUERC-59693	300		6825 ± 39	sieved peat	7642	[40]
7	Lagunas	-5.21	-75.64	126	Peru							3746	combine
		-5.21	-75.64	126	Peru	LA5		265	10	3646 ± 32	peat	3943	[19]
		-5.21	-75.64	126	Peru	LA4		285	10	3352 ± 32	peat	3549	[19]
8	Maquıía	-6.33	-74.81	117	Peru	MA9		415	10	2032 ± 22	peat	1959	[19]
9	Roca Fuerte	-4.44	-74.83	123	Peru	SL8		525	10	4505 ± 24	peat	5168	[19]
10	San Roque	-4.54	-74.63	119	Peru	SR5		555	10	6883 ± 33	peat	7696	[19]
11	Los Amigos Aguajal	-12.56	-70.12	240	Peru	LAA	Poz-119332	572		825 ± 30	bulk peat	709	[21]
12	Cuatro Vientos	-14.52	-61.12	169	Bolivia	CV	UGAMS 11809	229		5750 ± 30	bulk sediment	6529	[22]
13	Marahuaka I.2	3.67	-65.43	2684	Venezuela			40	40	5880 ± 50	sapric peat	6681	[23]
14	Huachama I.6	3.85	-65.73	1195	Venezuela			50		4840 ± 40	stemwood	5531	[23]
15	Cuao 2	5.05	-67.37	1060	Venezuela			71	32	3415 ± 30	sapric peat	[23]	[23]
16	Cuao 7	4.92	-67.18	936	Venezuela			50	20	3110 ± 35	sapric peat	3296	[23]
17	Cuao 11	4.98	-67.30	798	Venezuela			26.5	27	185 ± 30	hemic peat	179	[23]

18	Cuao 12	4.90	-67.35	1264	Venezuela			90	20	7030 ± 50	sapric peat	7845	[23]
19	Cuao 13	5.00	-67.43	632	Venezuela			40	10	105.3 ± 0.4	hemic peat	-60	[23]
20	Maigualida 1	5.57	-65.22	2119	Venezuela			120	20	6580 ± 50	sapric peat	7471	[23]
21	Maigualida 2	5.53	-65.15	1607	Venezuela			27.5	15	2530 ± 40	sapric peat	2588	[23]
22	Acopán-tepui	5.18	-62.08	2152	Venezuela							4123.5	combine
		5.18	-62.08	2152	Venezuela	ACO-2	WAT-1374	115	50	4380 ± 60	peat	4951	[24, 25]
		5.18	-62.08	2152	Venezuela	ACO-1	WAT-1380	170	40	3120 ± 80	peat	3296	[24, 25]
23	Auyán-tepui	5.92	-62.63	1353	Venezuela	AUY	WAT- 1678	143	50	4230 ± 80	peat	4726	[24, 25]
24	Churí-tepui	5.25	-62.02	2381	Venezuela	CHIM	WAT-1164	76	14	5740 ± 100	peat	6522	[24, 25]
25	Lake Encantada	4.70	-61.07	852	Venezuela							6090	combine
		4.70	-61.07	852	Venezuela	DV	WAT-1796	175	50	4260 ± 70	peat	4755	[24, 25]
		4.70	-61.07	852	Venezuela	PATAM4_D07	UCI - 37498	390.5	5	6530 ± 20	macrofossil	7425	[27]
26	Guaiquinima	5.90	-63.70	1465	Venezuela							6320.5	combine
		5.90	-63.70	1465	Venezuela	GUAIQ-2	WAT-1161	130	40	6000 ± 80	peat	6825	[24, 25]
		5.90	-63.70	1465	Venezuela	GUAIQ-1	WAT-1172	140	40	5100 ± 90	peat	5816	[24, 25]
27	Quebrada Arapán	5.17	-61.10	1102	Venezuela	ARA	WAT-1782	100	40	2600 ± 70	peat	2643	[24, 25]
28	Santa Cruz de Mapaurí	4.93	-61.10	866	Venezuela	MAP	WAT- 1787	210	60	8970 ± 90	peat	10044	[24, 25]
29	Santa Teresa pond	4.72	-61.08	856	Venezuela	ST	WAT- 1799	150	20	4040 ± 70	peat	4508	[24, 25]
30	Toronó-tepui	5.27	-62.15	2159	Venezuela							4392.5	combine

		5.27	-62.15	2159	Venezuela	TOR-2	WAT-1381	180	40	4040 ± 60	peat	4503	[24, 25]
		5.27	-62.15	2159	Venezuela	TOR-1	WAT-1370	125	50	3880 ± 60	peat	4282	[24, 25]
31	Urué	5.03	-61.17	852	Venezuela	UR	WAT-1928	180	40	1180 ± 70	peat	1071	[24, 25]
32	El Paují	4.47	-61.58	872	Venezuela	PATAM5_A07	Beta-251877	212.5	5	7280 ± 40	wood	8094	[26]
33	Laguna Las Margaritas	3.38	-73.43	265	Colombia		UtC-4957	592		5998 ± 41	bulk	6819	[41]
34	Mera	-1.48	-77.10	373	Ecuador	Mera-2	B-9618			33520 ± 1010	wood	38256	[33, 42, 43]
35	Nouragues	4.08	-52.67	193	French Guiana	NO 92-3	Beta-94330			2930 ± 110	microcharcoal	3064	[42-44]
36	Ogle Bridge	6.80	-58.15	3	Guyana		GrN-3506			>45000		46538	[42, 43, 45]
37	Kwakwani Canal	5.28	-58.05	31	Guyana		GrN-3103			6490 ± 80		7376	[42, 43, 45]
38	Torani Canal	5.82	-57.45	21	Guyana		GrN-3136			6140 ± 75		7005	[42, 43, 45]
39	River Courantyne	6.02	-57.63	17	Guyana		GrN-3109			6470 ± 85		7362	[42, 43, 45]
40	Yasuni	-0.45	-76.62	230	Ecuador	Maxus-1	NSRL-11685	225.5	1	250 ± 30	sediment	278	[28, 42, 43]
41	Yasuni	-0.45	-76.62	230	Ecuador	Maxus-4	NSRL-11186	245	2	4350 ± 35	wood	4907	[28, 42, 43]
42	Lake Gentry	-12.33	-68.87	248	Peru		NSRL-11998	43		940 ± 40	sediment	836	[35, 42, 43]
43	Lake Parker	-12.14	-69.02	246	Peru			50		525 ± 25	macrofossil	527	[35, 42, 43]
44	Ciega	3.07	-70.33	145	Colombia	200a	GrN-7065	565	10	14140 ± 120		17185	[34, 42, 43]
45	Laguna Huatacocha	-11.00	-75.00	975	Peru		WIS-1031			1100 ± 70		963	[42, 43, 46]

46	Laguna de Agua Sucia	3.50	-73.67	311	Colombia		GrN-3522	275	10	4110 ± 70	peat	4617	[47]
47	Chimantá massif	5.32	-62.23	2226	Venezuela	PATAM9-A07	Beta-242286	131	2	4900 ± 40	peat	5623	[29]
48	Cananguchal	-3.81	-70.23	92	Colombia	CAN	UBA-50266	193.5	1	603 ± 20	bulk peat (<63 μm)	606	this study
49	Caquetá	1.13	-75.43	212	Colombia	CAQ	UBA-50036	297.5	1	2021 ± 21	bulk peat (<63 μm)	1953	this study
50	Inírida	3.77	-67.93	99	Colombia	INI	UBA-50263	42.5	1	9825 ± 40	bulk peat (<63 μm)	11223	this study
51	Jenaro Herrera	-4.96	-73.67	135	Peru	JEN	SUERC-103538 (post bomb)	23.5	1	101.12 ± 0.46	bulk peat (<63 μm)	-6	this study
52	Puerto lleras	3.26	-73.42	241	Colombia	PLL	UBA-50040	298.5	1	3848 ± 25	bulk peat (<63 μm)	4228	this study

Supplementary Table 3 Detailed Information of study sites and the number of PyC samples.

Site	Lat (°)	Lon (°)	Core length (cm)	Core description	Core length included in the study (cm)	No. of PyC samples in peat section
Inírida (INI)	3.77	-67.93	100	Homogenous peat throughout the core.	42.5	5
Puerto Lleras (PLL)	3.26	-73.42	500	Homogeneous peat until 220 cm with clay intrusion between 20 and 38 cm; brown-grey sediment with peat stripes to 280 cm; dark peat with several clay/silt stripes from 280 to 500 cm.	300	11
Caquetá (CAQ)	1.13	-75.43	400	Peat until 50 cm; grey sediment between 50 and 120 cm; dark-brown peat with several thin grey stripes between 120 and 315 cm; grey sediment from 315 to 400 cm.	400	12
Quistococha (QUI)	-3.83	-73.32	300	Homogenous peat throughout the core.	300	16
Jenaro Herrera (JEN)	-4.96	-73.67	24	Homogenous peat throughout the core.	24	5
Cananguchal (CAN)	-3.81	-70.23	300	Homogeneous peat until 190 cm; gradual transition from peat to clay-rich sediment between 190 and 196 cm; yellowish clay materials from 196 to 300 cm.	200	18

Supplementary Table 4 Radiocarbon dates of study sites.

Site	Depth (cm)	Lab code	^{14}C age ± 1σ error	Calibrated range 2σ (cal. yr BP)
Inírida (INI)	20.5	UBA-51555	5,999 ± 39	6,679-6,941
	30.5	UBA-51964	8,480 ± 45	9,328-9,539
	42.5	UBA-50263	9,825 ± 40	11,174-11,268
	70.5	UBA-51556	5,664 ± 33	Exclude from model
	96.5	UBA-50264	9,697 ± 38	Exclude from model
Puerto lleras (PLL)	99.5	UBA-50038	2,388 ± 23	2,338-2,489
	198.5	UBA-50039	2,871 ± 24	2,875-3,064
	298.5	UBA-50040	3,848 ± 25	4,097-4,403
	400.5	UBA-50041	1,752 ± 22	Exclude from model
	499.5	UBA-50042	2,538 ± 31	Exclude from model
Caquetá (CAQ)	96.5	UBA-50034	263 ± 20	153-421
	202.5	UBA-50035	994 ± 21	797-928
	297.5	UBA-50036	2,021 ± 21	1,890-1,997
	398.5	UBA-50037	2,327 ± 27	2,155-2,359
Quistococha (QUI)	100.5	SUERC-103539	659 ± 37	554-661
	200.5	SUERC-103540	1,892 ± 35	1,711-1,878
	300	SUERC-103541	2,332 ± 37	2,151-2,425
Jenaro Herrera (JEN)	23.5	SUERC-103538	101.12 ± 0.46*	-69-6
Cananguchal (CAN)	111.5	UBA-50265	103 ± 20	31-258†
	193.5	UBA-50266	603 ± 20	540-632

* Values were reported as the fraction of modern carbon (%).

† Values were calibrated using IntCal20 due to the out of range in mixed calibration curve.

Supplementary Table 5 ^{210}Pb activity of Quistococha (QUI) and Jenaro Herrera (JEN) used in age-depth models.

**Site**	**Depth (cm)**	**Bulk density (g cm^{-3})**	**^{210}Pb (Bq kg^{-1})**	**^{210}Pb uncertainty**	**Calibrated age from *rplum* (cal. yr BP)**
QUI	0.5	0.0801	59.39	2.19	-68.0
QUI	1.5	0.0624	98.21	3.57	-66.3
QUI	2.5	0.0723	108.15	3.07	-64.1
QUI	3.5	0.0696	123.53	3.87	-61.2
QUI	4.5	0.0825	131.47	3.52	-57.4
QUI	5.5	0.0807	119.49	2.81	-52.6
QUI	6.5	0.0873	122.13	2.78	-46.9
QUI	7.5	0.0731	127.8	3.34	-40.3
QUI	8.5	0.0780	134.96	5.76	-34.3
QUI	9.5	0.0763	66.7	1.98	-28.9
QUI	10.5	0.0805	71.8	2.4	-23.6
QUI	11.5	0.0571	50.85	2.25	-18.5
QUI	12.5	0.0937	20.98	0.89	-14.6
QUI	13.5	0.0971	31.34	1.22	-11.9
QUI	14.5	0.0609	35.34	1.81	-8.6
QUI	15.5	0.0734	60.19	2.58	-4.7
QUI	16.5	0.1074	40.59	1.64	1.0
QUI	17.5	0.0734	29.69	1.88	8.4
QUI	18.5	0.0856	24.69	1.57	14.2
QUI	19.5	0.1039	10.73	0.91	18.5
QUI	20.5	0.0779	20.61	1.45	23.4
QUI	21.5	0.0529	19.53	1.74	28.9
QUI	22.5	0.0604	20.93	1.78	35.4
QUI	23.5	0.0414	23.02	1.56	42.7
QUI	24.5	0.0579	13.82	1.15	49.7
QUI	25.5	0.0570	14.78	0.98	56.6
QUI	26.5	0.0867	10.08	0.68	64.1
QUI	27.5	0.0712	10.87	0.85	72.3
QUI	28.5	0.0723	10.68	0.72	82.7
QUI	29.5	0.0627	9.68	0.8	95.3
QUI	30.5	0.0802	7.37	0.64	107.0
QUI	31.5	0.0600	22.56	1.08	117.9
QUI	32.5	0.0838	4.59	0.4	126.0
QUI	33.5	0.0820	4.51	0.44	131.4
QUI	34.5	0.0888	17.1	0.83	138.8
QUI	35.5	0.0631	10.45	0.7	148.3

QUI	36.5	0.0448	5.83	0.64	156.4
QUI	37.5	0.0909	5.73	0.45	163.1
QUI	38.5	0.0600	4.92	0.59	170.1
QUI	39.5	0.0842	6.66	0.48	177.4
JEN	0.5	0.1271	479.99	11.46	-67.6
JEN	1.5	0.1264	473.08	11.34	-65.2
JEN	2.5	0.1528	506.69	12.32	-62.2
JEN	3.5	0.1488	508.2	10.25	-58.5
JEN	4.5	0.1733	426.35	10.07	-54.4
JEN	5.5	0.1918	346.86	7.38	-50.1
JEN	6.5	0.1957	335.41	7.69	-45.5
JEN	7.5	0.2225	290.28	6.49	-40.2
JEN	8.5	0.2027	287.03	6.39	-34.3
JEN	9.5	0.2356	267.35	5.85	-27.1
JEN	10.5	0.2026	246.14	6.03	-18.8
JEN	11.5	0.2363	209.42	5.41	-9.0
JEN	12.5	0.2048	182.16	4.21	2.4
JEN	13.5	0.1912	113.69	2.88	12.8
JEN	14.5	0.1287	122.16	3.22	21.7
JEN	15.5	0.3582	70.69	3.15	32.0
JEN	16.5	0.2903	37.2	1.71	41.6
JEN	17.5	0.2286	45.09	2.04	49.7
JEN	18.5	0.1838	38.71	1.93	58.5
JEN	19.5	0.4149	26.67	1.87	67.3
JEN	20.5	0.2094	234.29	6.58	76.9
JEN	21.5	0.5281	15.44	0.84	86.6
JEN	22.5	0.4593	18.17	0.92	96.3
JEN	23.5	0.5155	31.39	1.63	105.6

Supplementary References:

1. Loisel, J., et al., *A database and synthesis of northern peatland soil properties and Holocene carbon and nitrogen accumulation.* The Holocene, 2014. **24**(9): p. 1028-1042.
2. Morris, P.J., et al., *Global peatland initiation driven by regionally asynchronous warming.* Proceedings of the National Academy of Sciences, 2018. **115**(19): p. 4851-4856.
3. Leifeld, J., et al., *Pyrogenic carbon contributes substantially to carbon storage in intact and degraded northern peatlands.* Land Degradation & Development, 2018. **29**(7): p. 2082-2091.

4. Schmidt, M.W., et al., *Comparative analysis of black carbon in soils.* Global Biogeochemical Cycles, 2001. **15**(1): p. 163-167.
5. Hammes, K., et al., *Comparison of quantification methods to measure fire‐derived (black/elemental) carbon in soils and sediments using reference materials from soil, water, sediment and the atmosphere.* Global Biogeochemical Cycles, 2007. **21**(3).
6. Meredith, W., et al., *Assessment of hydropyrolysis as a method for the quantification of black carbon using standard reference materials.* Geochimica et Cosmochimica Acta, 2012. **97**: p. 131-147.
7. Gustafsson, Ö., et al., *Quantification of the dilute sedimentary soot phase: Implications for PAH speciation and bioavailability.* Environmental Science & Technology, 1997. **31**(1): p. 203-209.
8. Honorio Coronado, E.N., et al., *Intensive field sampling increases the known extent of carbon-rich Amazonian peatland pole forests.* Environmental Research Letters, 2021. **16**(7): p. 074048.
9. Hastie, A., et al., *Risks to carbon storage from land-use change revealed by peat thickness maps of Peru.* Nature Geoscience, 2022. **15**(5): p. 369-374.
10. Melton, J.R., et al., *A map of global peatland extent created using machine learning (Peat-ML).* Geoscientific Model Development, 2022. **15**(12): p. 4709-4738.
11. Xu, J., et al., *PEATMAP: Refining estimates of global peatland distribution based on a meta-analysis.* Catena, 2018. **160**: p. 134-140.
12. Hastie, A., et al., *A new data-driven map predicts substantial undocumented peatland areas in Amazonia.* Environmental Research Letters, 2024. **19**(9): p. 094019.
13. Van Der Werf, G.R., et al., *Global fire emissions estimates during 1997-2016.* Earth System Science Data, 2017. **9**(2): p. 697-720.
14. Blaauw, M. and J.A. Christen, *Flexible paleoclimate age-depth models using an autoregressive gamma process.* 2011. **6**(3): p. 457-474.
15. Aquino-López, M.A., et al., *Bayesian analysis of 210 Pb dating.* Journal of Agricultural, Biological and Environmental Statistics, 2018. **23**(3): p. 317-333.
16. Jarvis, A., et al., *Hole-filled SRTM for the globe Version 4.* available from the CGIAR-CSI SRTM 90m Database (http://srtm. csi. cgiar. org), 2008. **15**(25-54): p. 5.
17. Lähteenoja, O., et al., *Amazonian peatlands: an ignored C sink and potential source.* Global Change Biology, 2009. **15**(9): p. 2311-2320.
18. Bhomia, R.K., et al., *Impacts of Mauritia flexuosa degradation on the carbon stocks of freshwater peatlands in the Pastaza-Marañón river basin of the Peruvian Amazon.* Mitigation and Adaptation Strategies for Global Change, 2019. **24**: p. 645-668.

19. Lähteenoja, O., et al., *The large Amazonian peatland carbon sink in the subsiding Pastaza‐Marañón foreland basin, Peru.* Global Change Biology, 2012. **18**(1): p. 164-178.
20. Draper, F.C., et al., *The distribution and amount of carbon in the largest peatland complex in Amazonia.* Environmental Research Letters, 2014. **9**(12): p. 124017.
21. Wang, B., et al., *Late Holocene peatland palm swamp (aguajal) development, carbon deposition and environment changes in the Madre de Dios region, southeastern Peru.* Palaeogeography, Palaeoclimatology, Palaeoecology, 2022. **594**: p. 110955.
22. Smith, R.J., et al., *Relating pollen representation to an evolving Amazonian landscape between the last glacial maximum and Late Holocene.* Quaternary Research, 2021. **99**: p. 63-79.
23. Zinck, J., P. García, and J. Van der Plicht, *Tepui Peatlands: age record and environmental changes.* Peatlands of the Western Guayana Highlands, Venezuela: Properties and Paleogeographic Significance of Peats, 2011: p. 189-236.
24. Schubert, C., P. Fritz, and R. Aravena, *Late quaternary paleoenvironmental studies in the Gran Sabana (Venezuelan Guayana shield).* Quaternary International, 1994. **21**: p. 81-90.
25. Medina, E., E. Cuevas, and O. Huber, *Origin of organic matter leading to peat formation in the southeastern Guayana uplands and highlands.* Peatlands of the Western Guayana Highlands, Venezuela: Properties and Paleogeographic Significance of Peats, 2011: p. 237-245.
26. Montoya, E., V. Rull, and S. Nogué, *Early human occupation and land use changes near the boundary of the Orinoco and the Amazon basins (SE Venezuela): Palynological evidence from El Paují record.* Palaeogeography, Palaeoclimatology, Palaeoecology, 2011. **310**(3-4): p. 413-426.
27. Montoya, E., et al., *Paleoecología del Holoceno en la Gran Sabana, SE Venezuela: Análisis preliminar de polen y microcarbones en la Laguna Encantada.* Collectanea Botanica, 2009. **28**: p. 65–79-65–79.
28. Weng, C., M.B. Bush, and J.S. Athens, *Holocene climate change and hydrarch succession in lowland Amazonian Ecuador.* Review of Palaeobotany and Palynology, 2002. **120**(1-2): p. 73-90.
29. Rull, V., et al., *Preliminary palynological analysis of a Holocene peat bog from Apakará-tepui (Chimantá Massif, Venezuelan Guayana).* Collectanea Botanica, 2011. **30**: p. 79-88.
30. Lähteenoja, O., B. Flores, and B. Nelson, *Tropical peat accumulation in Central Amazonia.* Wetlands, 2013. **33**: p. 495-503.

31. Crnobrna, B., et al., *Relationships between organic matter and bulk density in Amazonian peatland soils.* Sustainability, 2022. **14**(19): p. 12070.
32. Householder, J.E., et al., *Peatlands of the Madre de Dios River of Peru: distribution, geomorphology, and habitat diversity.* Wetlands, 2012. **32**: p. 359-368.
33. Bush, M.B., et al., *Late Pleistocene temperature depression and vegetation change in Ecuadorian Amazonia.* Quaternary Research, 1990. **34**(3): p. 330-345.
34. Van der Hammen, T., et al., *Glacial sequence and environmental history in the Sierra Nevada del Cocuy (Colombia).* Palaeogeography, Palaeoclimatology, Palaeoecology, 1980. **32**: p. 247-340.
35. Bush, M., M. Silman, and C. Listopad, *A regional study of Holocene climate change and human occupation in Peruvian Amazonia.* Analytical and Bioanalytical Chemistry, 2007. **34**(8): p. 1342-1356.
36. Santofimio Tamayo, G.A., *Carbon accumulation patterns in soils of tropical peatlands from alluvial origin (Caquetá, Colombia)*. 2018, Pontificia Universidad Javeriana: Bogotá, Colombia.
37. Roucoux, K.H., et al., *Vegetation development in an Amazonian peatland.* Palaeogeography, Palaeoclimatology, Palaeoecology, 2013. **374**: p. 242-255.
38. Kelly, T.J., et al., *Patterns and drivers of development in a west Amazonian peatland during the late Holocene.* Quaternary Science Reviews, 2020. **230**: p. 106168.
39. Kelly, T.J., et al., *The vegetation history of an Amazonian domed peatland.* Palaeogeography, Palaeoclimatology, Palaeoecology, 2017. **468**: p. 129-141.
40. Swindles, G.T., et al., *Ecosystem state shifts during long-term development of an Amazonian peatland.* Global Change Biology, 2018. **24**(2): p. 738-757.
41. Wille, M., et al., *Submillennium-scale migrations of the rainforest–savanna boundary in Colombia: 14C wiggle-matching and pollen analysis of core Las Margaritas.* Palaeogeography, Palaeoclimatology, Palaeoecology, 2003. **193**(2): p. 201-223.
42. Yu, Z., et al., *Global peatland dynamics since the Last Glacial Maximum.* Geophysical research letters, 2010. **37**(13).
43. Treat, C.C., et al., *Widespread global peatland establishment and persistence over the last 130,000 y.* Proceedings of the National Academy of Sciences, 2019. **116**(11): p. 4822-4827.
44. Ledru, M.-P., *Late holocene rainforest disturbance in French Guiana.* Review of Palaeobotany and Palynology, 2001. **115**(3-4): p. 161-170.

45. Van der Hammen, T., *A palynological study on the Quaternary of British Guiana.* Leidse Geologische Mededelingen, 1963. **29**(1): p. 125-168.
46. Wright, H., *Late Glacial and Late Holocene Moraines in the Cerros Cuchpanga, Central Peru1.* Quaternary Research, 1984. **21**(3): p. 275-285.
47. Wijmstra, T.A. and T. Van der Hammen, *Palynological data on the history of tropical savannas in northern South America.* Leidse Geologische Mededelingen, 1966. **38**(1): p. 71-83.
