# Supplementary material for: Wildfire legacies on pyrogenic carbon stocks in Amazonian peatlands
Source: Commun Earth Environ. 2025 Aug 19;6(1):678. doi: 10.1038/s43247-025-02674-7 (PMC12360956; doi:10.1038/s43247-025-02674-7)
Supplement: Supplementary file 3 — Reporting summary [file 43247_2025_2674_MOESM3_ESM.pdf]

nature portfolio

Corresponding author(s): Yuwan Wang, Ted R. Feldpausch

Last updated by author(s): Jul 28, 2025

Reporting Summary

Nature Portfolio wishes to improve the reproducibility of the work that we publish. This form provides structure for consistency and transparency in reporting. For further information on Nature Portfolio policies, see our Editorial Policies and the Editorial Policy Checklist.

Statistics

For all statistical analyses, confirm that the following items are present in the figure legend, table legend, main text, or Methods section.

n/a	Confirmed	
☐	☒	The exact sample size (*n*) for each experimental group/condition, given as a discrete number and unit of measurement
☐	☒	A statement on whether measurements were taken from distinct samples or whether the same sample was measured repeatedly
☐	☒	The statistical test(s) used AND whether they are one- or two-sided<br>*Only common tests should be described solely by name; describe more complex techniques in the Methods section.*
☐	☒	A description of all covariates tested
☐	☒	A description of any assumptions or corrections, such as tests of normality and adjustment for multiple comparisons
☐	☒	A full description of the statistical parameters including central tendency (e.g. means) or other basic estimates (e.g. regression coefficient) AND variation (e.g. standard deviation) or associated estimates of uncertainty (e.g. confidence intervals)
☐	☒	For null hypothesis testing, the test statistic (e.g. *F*, *t*, *r*) with confidence intervals, effect sizes, degrees of freedom and *P* value noted<br>*Give P values as exact values whenever suitable.*
☒	☐	For Bayesian analysis, information on the choice of priors and Markov chain Monte Carlo settings
☒	☐	For hierarchical and complex designs, identification of the appropriate level for tests and full reporting of outcomes
☒	☐	Estimates of effect sizes (e.g. Cohen's *d*, Pearson's *r*), indicating how they were calculated

Our web collection on statistics for biologists contains articles on many of the points above.

Software and code

Policy information about availability of computer code

Data collection	Data was not collected through code.
Data analysis	Spatial analysis were carried out in ArcGIS Pro 3.3.0 (Esri Inc.) and statistics analysis were computed using R (version 4.3.0) in Rstudio (2024.09.0+375).

For manuscripts utilizing custom algorithms or software that are central to the research but not yet described in published literature, software must be made available to editors and reviewers. We strongly encourage code deposition in a community repository (e.g. GitHub). See the Nature Portfolio guidelines for submitting code & software for further information.

Data

Policy information about availability of data

All manuscripts must include a data availability statement. This statement should provide the following information, where applicable:

- Accession codes, unique identifiers, or web links for publicly available datasets
- A description of any restrictions on data availability
- For clinical datasets or third party data, please ensure that the statement adheres to our policy

Data supporting the findings of this study are present in the paper and the Supplementary Materials. Datasets to generate the main figures are publicly available at https://doi.org/10.6084/m9.figshare.29643533.

Research involving human participants, their data, or biological material

Policy information about studies with human participants or human data. See also policy information about sex, gender (identity/presentation), and sexual orientation and race, ethnicity and racism.

Reporting on sex and gender	Not applicable
Reporting on race, ethnicity, or other socially relevant groupings	Not applicable
Population characteristics	Not applicable
Recruitment	Not applicable
Ethics oversight	Not applicable

Note that full information on the approval of the study protocol must also be provided in the manuscript.

Field-specific reporting

Please select the one below that is the best fit for your research. If you are not sure, read the appropriate sections before making your selection.

☐ Life sciences ☐ Behavioural & social sciences ☒ Ecological, evolutionary & environmental sciences

For a reference copy of the document with all sections, see nature.com/documents/nr-reporting-summary-flat.pdf

Ecological, evolutionary & environmental sciences study design

All studies must disclose on these points even when the disclosure is negative.

Study description	A total of six cores were collected from each of the lowland peatlands sites in Colombia (Inírida - INI, Puerto Lleras - PLL, Caquetá - CAQ and Cananguchal - CAN) and northern Peru (Quistococha - QUI and Jenaro Herrera - JEN) between 2018 and 2022. The cores were collected using a Russian D-section corer. All sites are permanently water-logged, and classified as Mauritia-dominated palm swamps (CAN, QUI and JEN), (mixed) palm swamps (PLL and CAQ) and tree-dominated swamps within flooded forests (INI). The study region is located in northwestern Amazonia, where the largest peat complex is described in the basin to date.
Research sample	Peat samples were sliced per 1 cm and sampled at 5-20 cm intervals depending on the total length of cores for pyrogenic carbon analysis.
Sampling strategy	Six peat cores were collected across northwestern Amazonia. The cores were distributed across the specific region to capture spatial variability, ensuring a comprehensive representation of peatland characteristics for analysis.
Data collection	Colombian peat cores:<br>Caquetá (CAQ) was sampled in March 2018; Belleza (BEL) was sampled in September 2018; Puerto Lleras (PLL) was sampled in February 2022; Cananguchal (CAN) was sampled in July 2022; Inírida (INI) was sampled in October 2022. Core collections and field trips were organised by Juan C. Benavides.<br><br>Peruvian peat cores:<br>Jenaro Herrera (JEN) and Quistococha (QUI) were sampled in October 2018. Core collections were organised by Euridice N. Honorio Coronado.
Timing and spatial scale	The sampling date was largely affected by the epidemic between 2020 and 2022 and sampling was suspended during the period.
Data exclusions	Sixty-seven of the 84 measured PyC values with a TOC >15% were included in the PyC-Age model analysis to represent the peat environment. This aligns with the assumption that TOC is evenly distributed across the age groups, which is based on the nature of peat formation. Unless there are exogenic mineral materials entering the system, TOC tends to present a relatively homogeneous profile with depth, like most of our cores. 15% of C is the lowest bound used for the classification of Amazon Basin peatland.<br><br>Excluded data belonged to CAQ and PLL, as these sites are heavily influenced by mineral material from river input, a situation that applies to sites near the Andes. Additionally, only one data point from the bottom of CAN (Mauritia-dominated palm swamps) was excluded, as CAN had not yet fully developed peat at that depth.
Reproducibility	Sufficient details are provided to reproduce our results.
Randomization	N/A
Blinding	N/A

Did the study involve field work? ☒ Yes ☐ No

Field work, collection and transport

Field conditions	Peat cores were extracted during dry season.
Location	The location of study sites is given in Table S3.
Access & import/export	All material collected, exported and imported follows the regulations of the country and relevant communities.
Disturbance	*Describe any disturbance caused by the study and how it was minimized.*

Reporting for specific materials, systems and methods

We require information from authors about some types of materials, experimental systems and methods used in many studies. Here, indicate whether each material, system or method listed is relevant to your study. If you are not sure if a list item applies to your research, read the appropriate section before selecting a response.

Materials & experimental systems

n/a	Involved in the study
☒	☐ Antibodies
☒	☐ Eukaryotic cell lines
☒	☐ Palaeontology and archaeology
☒	☐ Animals and other organisms
☒	☐ Clinical data
☒	☐ Dual use research of concern
☒	☐ Plants

Methods

n/a	Involved in the study
☒	☐ ChIP-seq
☒	☐ Flow cytometry
☒	☐ MRI-based neuroimaging

Plants

Seed stocks	*Report on the source of all seed stocks or other plant material used. If applicable, state the seed stock centre and catalogue number. If plant specimens were collected from the field, describe the collection location, date and sampling procedures.*
Novel plant genotypes	*Describe the methods by which all novel plant genotypes were produced. This includes those generated by transgenic approaches, gene editing, chemical/radiation-based mutagenesis and hybridization. For transgenic lines, describe the transformation method, the number of independent lines analyzed and the generation upon which experiments were performed. For gene-edited lines, describe the editor used, the endogenous sequence targeted for editing, the targeting guide RNA sequence (if applicable) and how the editor was applied.*
Authentication	*Describe any authentication procedures for each seed stock used or novel genotype generated. Describe any experiments used to assess the effect of a mutation and, where applicable, how potential secondary effects (e.g. second site T-DNA insertions, mosiacism, off-target gene editing) were examined.*
